# A genome-scale metabolic network reconstruction of extremely halophilic bacterium *Salinibacter ruber*

**DOI:** 10.1371/journal.pone.0216336

**Published:** 2019-05-09

**Authors:** Maryam Bagheri, Sayed-Amir Marashi, Mohammad Ali Amoozegar

**Affiliations:** 1 Extremophiles Laboratory, Department of Microbiology, School of Biology and Center of Excellence in Phylogeny of Living Organisms, College of Science, University of Tehran, Tehran, Iran; 2 Department of Biotechnology, College of Science, University of Tehran, Tehran, Iran; Osmania University, INDIA

## Abstract

A genome-scale metabolic network reconstruction of *Salinibacter ruber* DSM13855 is presented here. To our knowledge, this is the first metabolic model of an organism in the phylum *Rhodothermaeota*. This model, which will be called *i*MB631, was reconstructed based on genomic and biochemical data available on the strain *Salinibacter ruber* DSM13855. This network consists of 1459 reactions, 1363 metabolites and 631 genes. Model evaluation was performed based on existing biochemical data in the literature and also by performing laboratory experiments. For growth on different carbon sources, we show that *i*MB631 is able to correctly predict the growth in 91% of cases where growth has been observed experimentally and 83% of conditions in which *S*. *ruber* did not grow. The F-score was 93%, demonstrating a generally acceptable performance of the model. Based on the predicted flux distributions, we found that under certain autotrophic condition, a reductive tricarboxylic acid cycle (rTCA) has fluxes in all necessary reactions to support autotrophic growth. To include special metabolites of the bacterium, salinixanthin biosynthesis pathway was modeled based on the pathway proposed recently. For years, main glucose consumption pathway has been under debates in *S*. *ruber*. Using flux balance analysis, *i*MB631 predicts pentose phosphate pathway, rather than glycolysis, as the active glucose consumption method in the *S*. *ruber*.

## Introduction

### Special characteristics of *Salinibacter ruber* and its metabolism

*Salinibacter* is the type genus of *Salinibacteriaceae*, a recently described family of halophilic bacteria, living under extreme conditions of hypersaline brines with a high concentration of salt, up to saturation [1, 2]. *Salinibacter ruber* was the first bacterium shown to be active at hypersaline brines [3–5]. Prior to its discovery, the archaeal family *Halobactreiaceae* were considered to be the only prokaryotes active in hypersaline brines [3, 4].

Soon after its description, *S*. *ruber* attracted intensive attention because of its exceptional characteristics. Despites phylogenetic affiliation of *S*. *ruber* to bacteria, it has many features in common with members of archaeal family *Halobactreiaceae* [6]. The most interesting feature of *S*. *ruber* is its haloadaptation mechanism [7] which has been shown to be the archaeal type of haloadaptation (*i*.*e*., the “salt-in” strategy) which is the intracellular accumulation of mineral ions, mostly K^+^ and Cl^-^. The salt-in strategy affects the whole proteome structure and goes along with extensive genome wide adjustments to prepare the whole enzymatic system to be functional under hypersaline cytoplasmic conditions [8, 9].

The haloadaptation mechanism of *S*. *ruber* was studied based on sequencing and annotating its genome [10]. By analyzing the (predicted) proteome of this bacterium, it was suggested that the main origin of the proteome is of bacterial, and the archaeal-like properties, including salt-dependent activities of some enzymes, amino acid composition and the isoelectric point of bulk proteins, have been mainly acquired through convergent evolution [6, 10, 11].

*Salinibacter ruber* has interesting metabolic characteristics including production of retinal pigments as chloride pump halorhodopsin (HR) and light-driven proton pump, xanthorhodopsin. Furthermore, this bacterium specifically produces certain metabolites including halocapnine derivatives and salinixanthin [12–19].

### *Genome*-scale metabolic reconstructions

Sequencing, biochemical, physiological and “omics” data available for a certain organism can be systematized, integrated and mathematically formulated in a well-defined genome-scale metabolic model (GEM). Such a reconstruction is a useful knowledge-base for numerous applications, including modeling and understanding biological communities and interactions, genetic engineering, shedding light to evolutionary traits, and comparative analysis [20–24]. Though GEMs have been developed for a variety of organisms over all three domains of life, the availability of such systems is not well distributed over all physiological types of organisms. Well-known organisms are studied more often and with improved details while exceptional ones like extremophiles are rarely considered.

Extremely halophilic microorganisms are among the least metabolically studied species, to the best of our knowledge. Previously, GEMs of only two archaeal extreme halophiles have been previously reconstructed, namely, *Halobacterium salinarum* [25] and *Natronomonas pharaonis* [26]. On the other hand, a holistic understanding of metabolic pathways in extremely halophilic bacteria is yet to be presented. In this work, we describe the reconstruction of a detailed GEM for *Salinibacter ruber*, the first reconstructed GEM for members of the bacterial phylum *Rhodothermaeota* and also the first extremely halophilic bacterial GEM reconstructed to date. Our aim is to shed light on the unusual metabolism and salt adaptation mechanism of *S*. *ruber* using the systemic approach of GEM.

## Materials and methods

### Reconstruction of metabolic network

For genome-scale metabolic network reconstruction, the genomic sequence of *S*. *ruber* DSM13855^T^ was obtained from GenBank under accession numbers CP000159 and CP000160 (chromosome and plasmid respectively). These genomic sequences were re-annotated using RAST [27] and KAAS [28] servers to get compatible with ModelSEED server and KEGG database. Three draft metabolic models were obtained separately using the ModelSEED [29], MetaCyc [30] and KEGG, based on the original and re-annotated genomes. Reaction and metabolite ids of these networks where then changed to the best matching ModelSEED ids to make them comparable. This was done by means of biochemistry database file provided by ModelSEED and also by using MetaNetX [31]. In cases were no equivalent for an id was found, personalized ids were defined to mark the corresponding metabolites or reactions. These draft networks were then compared together and curated manually by editing, adding and removing reactions based on the biochemical information about *S*. *ruber* available in the literature and also public databases, including BRENDA [32], KEGG [33], UniProt [32] and BioCyc [30]. Duplicated or triplicated reactions from three draft models were unified and other reactions which were not present in all three models were considered manually whether to be added or deleted in the final model. Reaction directionality and stoichiometry was also checked using ModelSEED, BiGG [34], Rhea [35] and BioCyc [30]. Gene association of each reaction was improved first by manual comparison of annotations from KAAS, RAST and the published annotation on GenBank and then by checking the relation of proposed gene(s) to each reaction. KEGG reaction ID, subsystem relation, protein KO and EC number were added to the model manually using KEGG REST API (Representational State Transfer Application Programming Interface). Some reactions were defined manually to best fit the biochemistry of *Salinibacter*. Salinixanthin biosynthesis has the best examples of these kind of reactions. The biosynthesis mechanism of this compound was based on the pathway proposed by Ron et al [36]. Some reactions and metabolites of this pathway are not represented in KEGG, ModelSEED or MetaCyc. New ids were defined and attributed with known properties of each.

In the next stage, COBRA toolbox [37, 38] was used to improve the network, reconstruct the mathematical model and run FBA analysis. Gaps were detected using functions “detectDeadEnds” and “GapFind”. Manual gap-filling was also performed by mapping reactions to MetaCyc and KEGG databases. Gap filling reactions were obtained from both KEGG and ModelSEED databases and added to the model. Memote online software was used to evaluate the sbml formatted model file [39]. SBO terms and Gene ontology were added to the model manually. Metabolite and reaction annotations, mass and charge balances, annotation conformity and curation of orphan metabolites were further improved by considering memote reports and manual corrections wherever possible.

### Biomass composition

The biomass composition, which shows the main constituent of dried cell biomass, is typically included in metabolic network models to show their accumulation. Precisely, these metabolites can be considered as reactants of an imaginary reaction, called biomass production. The flux of this reaction, which is a proxy of growth rate, can be used as the objective function in FBA.

No detailed information on the biomass composition of *S*. *ruber* has been published. Here, we defined the biomass composition by integrating data from different literatures referenced here with those taken from the published composition of *Escherichia coli* model, iAF1260 with some manual adjustments [5, 11, 40–44].

### Flux balance analysis

Based on steady-state assumption, the concentrations of the cellular metabolites remains constant during the analysis. Flux balance analysis (FBA), a widely used computational method, is based on this assumption. FBA finds a metabolic flux distribution in steady-state that maximizes a defined objective, *e*. *g*., biomass production rate, *v*_biomass_ [45]. Briefly, FBA is formulated as a linear programming problem;

$$Maximize\;Z\;=\;v_{biomass}$$

SubjecttoS.v=0

$$v_{i,\;min}\;\leq v_{i}\;\leq \;v_{i,\;max}\;(for\;i\;=\;1,\;\ldots ,\;n)$$

Where ***Z*** denotes the objective function, C is a row vector showing the influence of individual fluxes on the objective function, ***v*** is the reaction flux vector, n denotes the number of reactions. V_i, min_ and V_i, max_ are the lower and upper bounds of the flux V_i_. Here, FBA was performed using COBRAToolbox-2.0, with the ‘‘optimizeCbModel” function to solve FBA problems using glpk solver version 4.47.

### Carbon source utilization

A frozen stock of *S*. *ruber* DSM13855 was used to prepare preculture, inoculating 100-ml Erlenmeyer flasks containing 50 ml modified growth medium (MGM) with 23% total salt [46]. This medium contains a 23% salt mixture prepared from a 30% stock solution, which consists of (per liter): 240 g NaCl, 35 g MgSO_4_, 30 g MgCl_2_, 7 g KCl and 1 g CaCl_2_, supplemented with 1% (w/v) peptone and 0.2% (w/v) yeast extract. The pH of the medium was adjusted to 7.2–7.4 with Tris-base (Merck). Carbon source utilization (1%, w/v) was tested by omission of peptone from MGM broth and reduction of yeast extract concentration to 0.1 g l^-1^ [47]. The ability of the strain to grow anaerobically in the presence of 5.0 g l^-1^ nitrate, DMSO and arginine (fermentation) was tested in MGM broth prepared anaerobically in serum tubes according to the procedures described in references [47–49].

### Model evaluation

Evaluation of the model was performed using available data on metabolism of *S*. *ruber* DSM13855 in the scientific literature. Briefly, we collected experimental data such as amino acid and carbohydrate utilization from literature. Subsequently, for each case, the minimum and maximum limits of exchange reactions were defined to simulate the growth conditions in each case. The predictions of the model were then compared to laboratory observations. Inconsistencies were checked manually and corrected for adding, removing or changing corresponding reactions whenever possible.

## Results

### General characteristics of the reconstructed GEM of *Salinibacter ruber* DSM13855

The *i*MB631 model (S1 and S2 Files) consists of 1459 reactions, 1363 metabolites and 631 genes (S3 File). Overall 21.8% of the 2894 genes described in the genome annotation, which comprises 32% of non-hypothetical genes [10] were included in the model. In our model 85.26% of the reactions are associated to at least one gene. The model includes 65 transport reactions, 61 exchange and 1394 metabolic reactions. Table 1 compares general characteristics of *i*MB631 to models of extremely halophilic archaeon *Halobacterium salinarum* and moderately halophilic bacterium *Chromohalobacter salexigens*, *i*OG490 and *i*OA584 respectively [25, 50]. From the table it can be conferred that, comparing to these to halophilic models, *i*MB631 has good and acceptable features in light of general statistics.

**Table 1:** Comparison of general characteristics of *i*MB631 with previously reconstructed networks of halophiles, *i*OA584 and *i*OG490 representatives of bacterial and archaeal halophilic models respectively.

	*i*MB631	*i*OA584	*i*OG490
Total Genes in Genome	2894	3412	2867
Included number of genes	631	584	490
Total number of reactions	1459	1386	711
Exchange	61	500	59
transport	65	31	52
Non-genes associated	216	510	113
Total Metabolites	1363	1411	557
Intracellular metabolites	1301	920	
Extracellular metabolites	62	491	

The model genes are 53% monofunctional where the rest of the genes are multifunctional, conducting more than one reaction in the model. Taking connectivity as the number of reactions in which a metabolite participates, the *i*MB631 model presents 554 out of the 1363 metabolites participate in two metabolic reactions, 143 in three reactions, and 240 in more than three reactions. By tuning the exchange reactions to reflect the condition of MGM medium in FBA, the model predicted a growth rate of 0.297 h^-1^.

### Model validation

The most important outcome in reconstructing a model, is its ability to predict the physiology of the respective organism. FBA results showed that *S*. *ruber* can utilize citrate, acetate, alanine, threonine, serine and glycine but not valine, lysine and leucine. Anaerobic respiration was negative in the presence of all of the three tested compounds, namely, 5.0 g l^-1^ nitrate, DMSO and arginine. *In silico* analysis using *i*MB631 predicted positive growth by utilizing citrate, acetate, alanine, threonine, serine, glycine and valine and negative growth results on lysine and leucine. Table 2 summarizes the *in silico vs* experimental results. Except for valine, the model predictions are in accordance with observed phenotypic results. These results indicate an acceptable prediction power for *i*MB631. Laboratory tests of catalase activity, Starch hydrolysis, DNA hydrolysis, Gelatin hydrolysis, Indole production, utilization of glycerol, glucose, maltose, fructose, galactose, arabinose, ribose, xylose, sucrose, lactose, cellobiose, raffinose, L-tryptophan, L-asparagine, L-arginine, L-methionine, L-tyrosine, L-cysteine and L-aspartate which were previously reported for the strain were simulated in our model. Table 3 summarizes the comparison of results of simulations using *i*MB631 and previously published data. Biomass was optimized in simulated MH medium at 0.0291 mmol/gDW/h.

**Table 2:** Comparison of experimental and predicted behavior of *S*. *ruber* DSM13855.

Utilization source	Experimental results	*In silico* results	results
**citrate**	+	+	✓
**acetate**	+	+	✓
**alanine**	+	+	✓
**threonine**	+	+	✓
**serine**	+	+	✓
**glycine**	+	+	✓
**valine**	-	+	×
**lysine**	-	-	✓
**leucine**	-	-	✓
**Anaerobic growth on arginine**	-	-	✓
**Anaerobic growth on DMSO**	-	-	✓
**Anaerobic growth on nitrate**	-	-	✓

**Table 3:** Comparison of previously published phenotypes of *S*. *ruber* DSM13855and predicted behavior of *i*MB631.

Utilization source	Experimental results	*In silico* results	results	reference
**Catalase activity**	+	+	✓	[44]
**Starch hydrolysis**	+	+	✓	[44]
**DNA hydrolysis**	+	+	✓	[44]
**Gelatin hydrolysis**	+	+	✓	[44]
**Indole production**	+	+	✓	[44]
**glycerol**	+	+	✓	[51]
**glucose**	+	+	✓	[44]
**maltose**	+	+	✓	[44]
**fructose**	-	+	×	[52]
**galactose**	-	-	✓	[44]
**arabinose**	-	-	✓	[44]
**ribose**	-	-	✓	[52]
**xylose**	-	-	✓	[52]
**sucrose**	-	+	×	[44]
**lactose**	-	-	✓	[44]
**cellobiose**	-	+	×	[52]
**raffinose**	-	-	✓	[44]
**L-tryptophan**	+	-	×	[52]
**L-asparagine**	-	-	✓	[44]
**L-arginine**	+	+	✓	[44]
**L-methionine**	+	+	✓	[44]
**L-tyrosine**	+	+	✓	[44]
**L-cysteine**	+	+	✓	[44]
**L-aspartate**	-	+	×	[44]

Using both experimental results and available published literature to validate the predictions of *i*MB631, this model was able to correctly predict the growth in 91% of cases where growth has been observed experimentally and 83% of conditions in which *S*. *ruber* did not grow.

### Case analysis of Embden–Meyerhof glycolytic and pentose phosphate pathways

Metabolic pathway by which glucose is consumed in *Salinibacter ruber* has been under debate. As Oren states in 2013; “Activities of a constitutive, salt-inhibited hexokinase and a constitutive, salt-dependent NADP-linked glucose-6-phosphate dehydrogenase were found, but fructose-1, 6-bisphosphate aldolase activity was not detected. Glucose degradation by the Entner–Doudoroff pathway was therefore suggested, but no activity of 2-keto-3-deoxy-6-phosphogluconate aldolase was found [52]. Genomic analysis later showed genes for a complete Embden–Meyerhof glycolytic pathway [10].[7]

**Fig 1:**
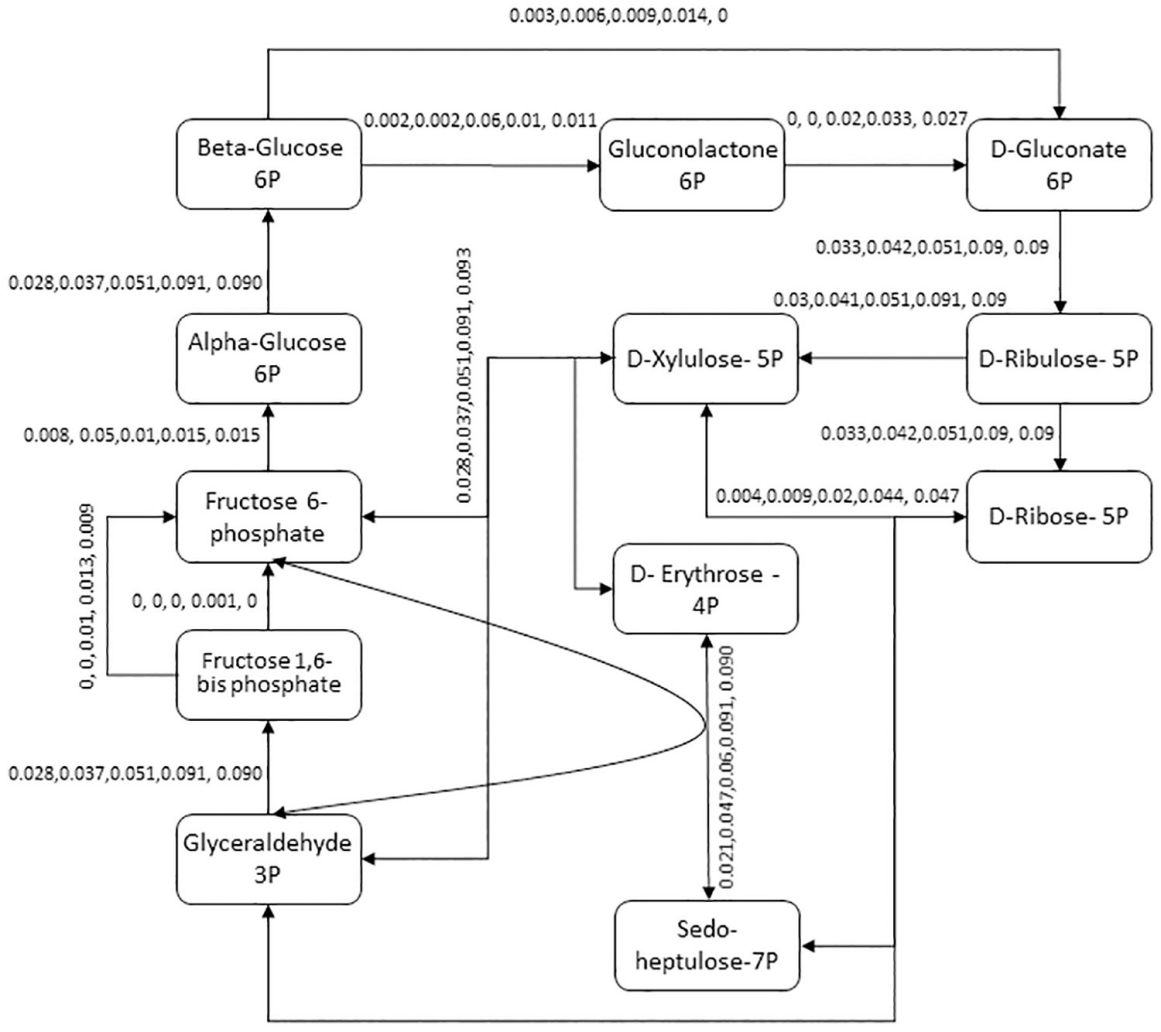
Pentose phosphate pathway in *i*MB631. For simplification, cofactors and byproducts are not shown. Each reaction’s flux is shown for glucose uptake rates of 0.5, 1, 2, 4 and 5 mmol/gDW/h. The cycle works in all 5 uptake rates. Though glycolysis pathway is presented in the model, no glucose uptake rate was found where all reactions have acceptable fluxes.

Through our model reconstruction, it was found all necessary genes for both glycolysis and pentose phosphate pathways. Reactions related to both pathways were added to the model. Using FBA analysis, we tried to describe the fluxes through both pathways’ reactions. Glucose uptake rates of 0.5, 1, 2, 4 and 5 mmol/gDW/h were checked. In none of these cases, all necessary Embden–Meyerhof glycolytic pathway reactions had none-zero fluxes to produce pyruvate from glucose. Reactions corresponding to KEGG reaction ids R01518, R01662, R01786 and R04779 were carrying zero fluxes at different glucose uptake rates, resulting in zero or unfeasibly low production of pyruvate from glucose by Embden–Meyerhof glycolytic pathway. Biomass production was 0.006, 0.091, 0.013, 0.016 and 0.015 for glucose uptake rates of 0.5, 1, 2, 4 and 5 mmol/gDW/h respectively. Glucose was consumed through pentose phosphate pathway. Metabolic fluxes for pentose phosphate pathway are highlighted in Fig 1.

### Bioenergetics of *Salinibacter ruber*

Heterotrophic lifestyle and aerobic growth of *Salinibacter ruber* were analyzed as representatives of the most important parts of its bioenergetics. *Salinibacter ruber* has long been reported as a strictly aerobic and heterotrophic bacterium. Based on predicted genes in the genome, *i*MB631 predicts the bacterial respiratory physiology to be strictly aerobic using both clusters of cytochrome c oxidase presented in the genome. On the other hand, considering the organic carbon demand, an unexpected ability to use reductive tricarboxylic acid cycle for an autotrophic life style. Key enzymes of the pathway are present in the pathway except for citryl-CoA synthetase. Because no experimental data is available to support autotrophic growth in *S*. *ruber*, the reactions were omitted from the final model.

Oxidative phosphorylation mechanism was also investigated in the bacterium. *S*. *ruber* seems to have a complete gene set related to NADH dehydrogenase complex. Genes related to complex III analogue were not found in the bacterium.

**Fig 2:**
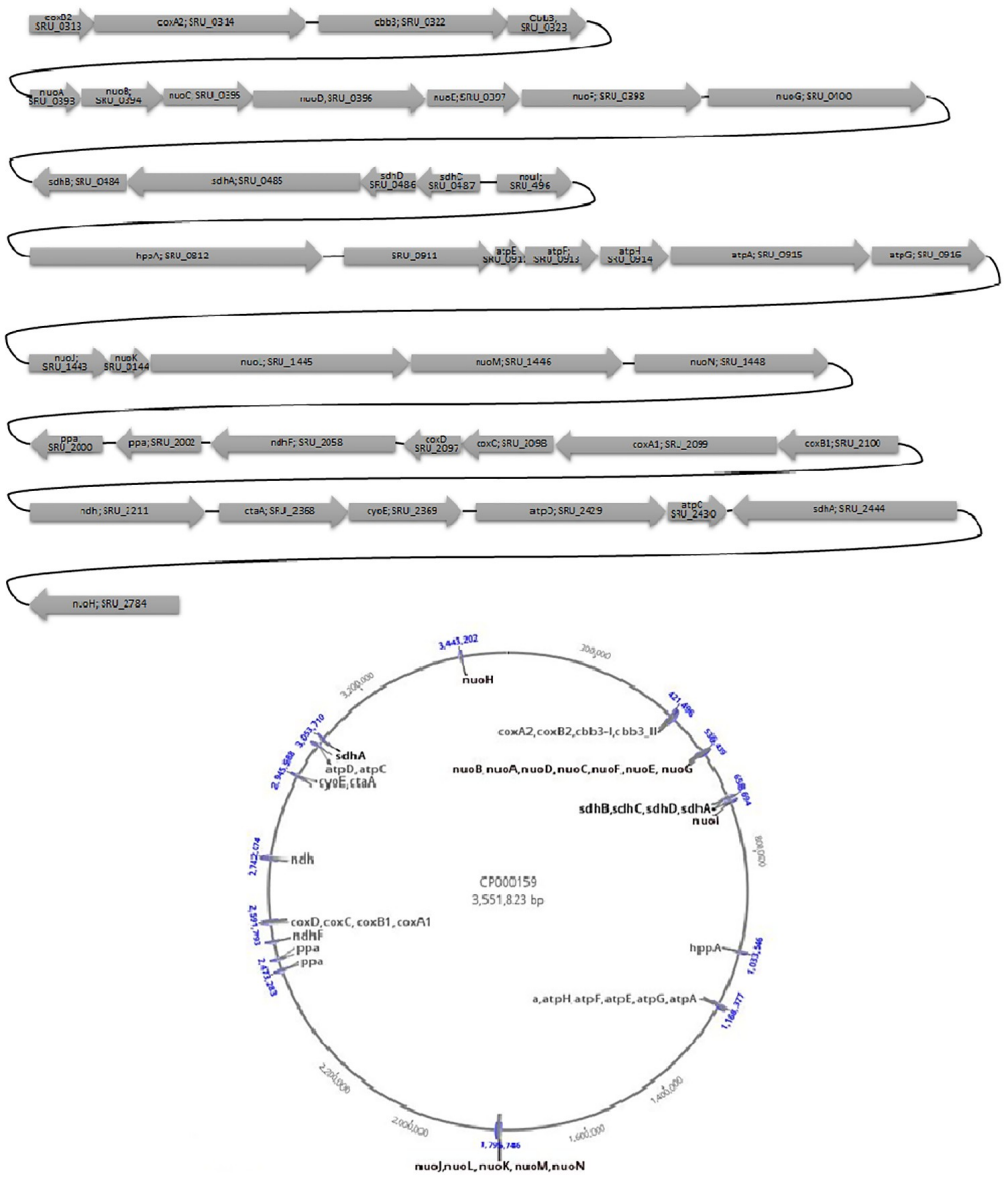
Phosphorylation oxidative genes in *S*. *ruber*. (A). gene direction and related position of their related position to each other. (B). distribution of gene clusters in the genome. Totally 42 genes were related to oxidative phosphorylation reactions which are localized at 14 segments of the main chromosome.

The genes related to oxidative phosphorylation pathway for *S*. *ruber* are shown in Fig 2. Menaquinone 7 (MK-7) was previously reported to be the only respiratory quinone present in *Salinibacter* [5, 44]. In contrast to *H*. *salinarum*, *S*. *ruber* seems to have a complete gene set related to NADH dehydrogenase complex. In both extremely halophilic strains, menaquinone, rather than ubiquinone has been proposed to shuttle electrons from complex I to Complex III analogue. Genes related to complex III analogue were not found in the *S*. *ruber*. Instead of cytochrome c1 subunit (*petC*), an archaeal blue copper protein (halocyanin) was previously proposed to play the carrier role in *H*. *salinarum*. Mongoldin et al showed that *Salinibacter* genome has two clusters of cytochrome c oxidase subunit I and II genes, with coxA1 and coxB1 (SRU2099 and SRU2100) and coxA2 and coxB2 (SRU0314 and SRU0313) which coxB2 and coxA2 are most closely related to their haloarchaeal homologs [10] and are located apart from other cytochrome c oxidase subunite genes, namely coxC and coxD. Though some genes related to anaerobic respiration on nitrite and sulfur compounds have been detected in the genome of *S*. *ruber* and related reactions were included in the model, no considerable biomass production was achieved when anaerobic growth conditions where simulated. Fluxes in all related reactions remained almost zero in different anaerobic simulations performed.

The reverse tricarboxylic acid or reductive tricarboxylic acid (rTCA) cycle is a carbon fixation cycle that synthesize an acetyl CoA by moving the TCA cycle backward, using 2 molecules of CO_2_, 8 NADH and/or FADH, and 2 ATP [53]. *Chlorobium* species, certain sulfate reducing deltaproteobacteria like *Desulfobacter hydrogenophilus*, some thermophilic *Aquificales* (*Aquifex* and *Hydrogenobacter thermophiles*) and archaea of the family *Thermoproteaceae* like *Thermoproteus* have been shown to use reverse tricarboxylic acid cycle [54, 55].

**Fig 3:**
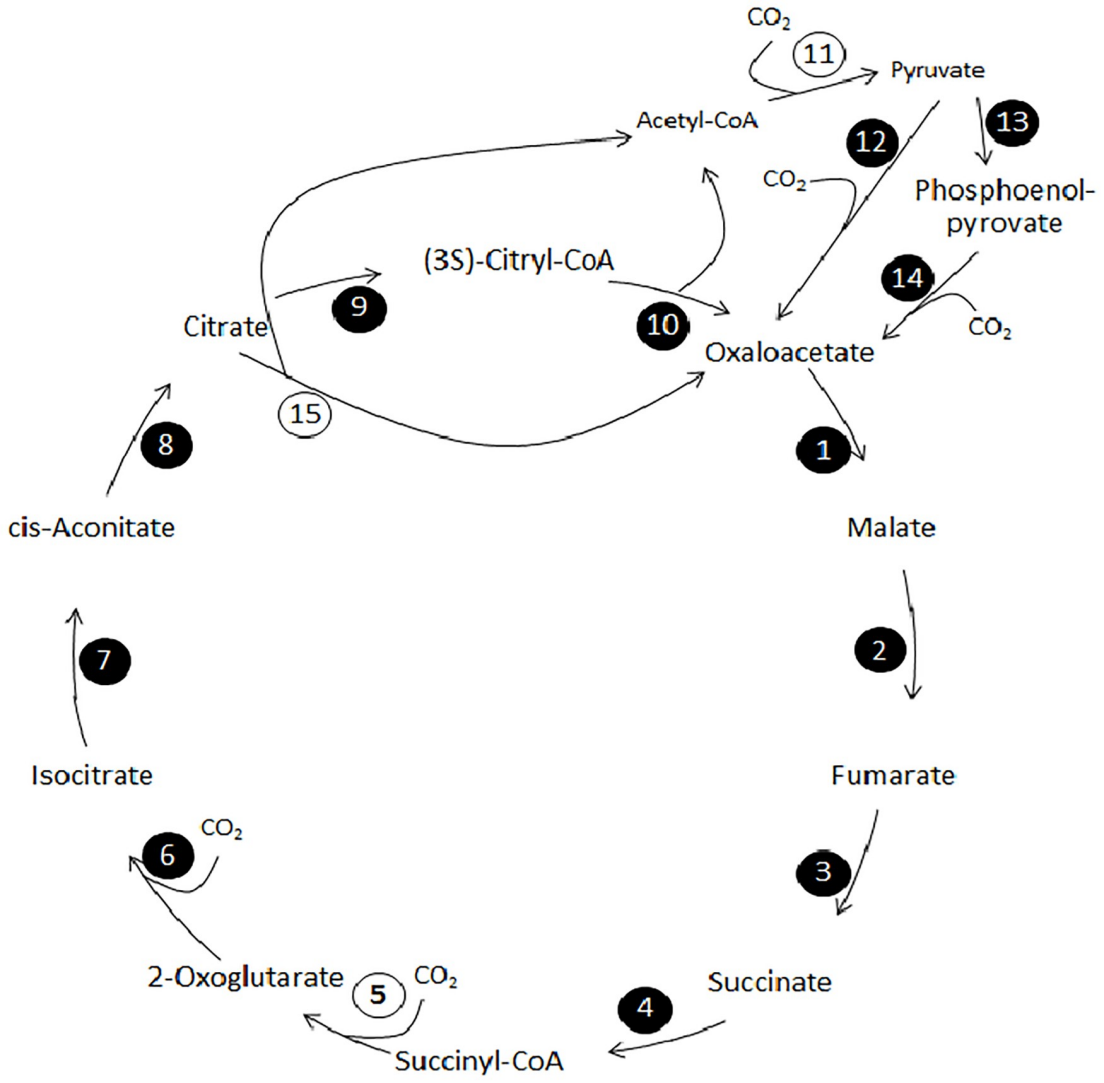
Schematic representation of rTCA modified from KEGG map; map00720. Reactions are coded by numbers in circles and described in Table 4. Critical reactions of the pathway i. e. those which are conducted by key enzymes are shown with numbers in white circles wherease others are circled in black. Comparison of the nessesary reactions of the pathway with those present in *Salinibacter ruber* DSM13855, suggests that this pathway can be present in *Salinibacter*’s metabolism. When these reactions where included in the reconstructed model, FBA analysis showed that it can carry out fluxes under low nutrition conditions.

Originally discovered in *Chlorobium*, a green sulfur phototrophic bacterium, rTCA cycle has many reactions that are TCA ones, just running in reverse direction but there are key enzymes that catalyze energy demanding and critical reactions and make the reversal of the TCA cycle possible. Three Key enzymes have been identified to catalyze the energetically unfavorable reactions and make the rTCA cycle practical, namely, ATP citrate synthase, pyruvate synthase and 2-oxoglutarate synthase [54]. Fig 3 shows a schematic view of rTCA cycle and the position of critical enzyme. Conversion of citrate to citryl-CoA by the enzyme citrate-CoA ligase and then to oxaloacetate by citryl-CoA lyase is a substitution to ATP citrate synthase reaction.

A summary of genes and reactions of the cycle in *S*. *ruber* is presented in Table 4. Except for ATP citrate synthase or alternatively citrate-CoA ligase, all related enzyme’s genes are presented in the genome. Two of three key enzymes of the pathway, namely pyruvate synthase and 2-oxoglutarate synthase are present and adding the reaction related to citrate-CoA ligase to the model, will make the pathway flux active at simulated autotrophic environment by a optimal biomass production rate of 0.0164. Gap-filling the model by ATP citrate synthase instead of citrate-CoA ligase, reduces the biomass production of the model in autotrophic condition to near zero.

**Table 4:** Description of reactions of rTCA and their related genes and enzymes in *S*. *ruber* DSM13855.

	Kegg ID	Enzyme recommended name in Brenda (*EC)*	Gene in *S*.*ruber*	reaction details	notes
1	R00342	malate dehydrogenase (1.1.1.37)	SRU_1571	Oxaloacetate + NADH + H^+^ ↔ NAD^+^ + L − Malate	
2	R01082	fumarate hydratase (4.2.1.2)	SRU_1611	L − Malate ↔ H_2_O + Fumarate	
3	R02164	succinate dehydrogenase / fumarate reductase (1.3.5.1, 1.3.5.4)	SRU_0485 SRU_2444 SRU_0484	Fumarate + QH_2_ ↔ Succinate + Q	
4	R00405	succinate-CoA ligase ADP-forming (6.2.1.5)	SRU_0670 SRU_1125	ATP + CoA + Succinate ↔ ADP + Phosphate + Succinyl − CoA	
5	R01197	2-oxoglutarate synthase (1.2.7.3)	SRU_0424	CO_2_ + H^+^ + Succinyl − CoA + 2 Reduced ferredoxin ↔ CoA + 2 − Oxoglutarate + 2 Oxidized ferredoxin	Key Enzyme
6	R00267	isocitrate dehydrogenase (NADP+) (1.1.1.42)	SRU_1973	NADPH + CO_2_ + 2 − Oxoglutarate ↔ NADP + Isocitrate	
7	R01900	aconitate hydratase (4.2.1.3)	SRU_1866 SRU_1477	Isocitrate ↔ H_2_O + cis − Aconitate	
8	R01325	aconitate hydratase (4.2.1.3)	SRU_1866 SRU_1477	H_2_O + cis − Aconitate ↔ Citrate	
9	R01322	citrate-CoA ligase (6.2.1.18)	Not found	Citrate + ATP + CoA + H^+^ ↔ ADP + Phosphate + (3S) − Citryl − CoA	with R00354 substitutes key reaction R01196
10	R00354	citryl-CoA lyase (4.1.3.34)	SRU_1685	(3S) − Citryl − CoA ↔ Acetyl − CoA + Oxaloacetate + H^+^	with R00322 substitutes key reaction R01196
11	R01196	pyruvate synthase (1.2.7.1)	SRU_0423	CO_2_ + Acetyl − CoA + H^+^ + 2 Reduced ferredoxin ↔ CoA + Pyruvate + 2 Oxidizedferredoxin	Key Enzyme
12	R00344	pyruvate carboxylase (6.4.1.1)	SRU_0828	ATP + Pyruvate + H_2_2CO_3_ ↔ ADP + Phosphate + Oxaloacetate + H^+^	
13	R00199	pyruvate,water dikinase (2.7.9.2)	SRU_1138	H_2_O + ATP + Pyruvate ↔ Phosphate + AMP + Phosphoenolpyruvate + 3H^+^	
14	R00345	phosphoenolpyruvate carboxylase (4.1.1.31)	SRU_0093	H_2_O + CO_2_ + Phosphoenolpyruvate ↔ Phosphate + Oxaloacetate + H^+^	

It should be noted that almost all enzymes of the rTCA cycle can be present in non-autotrophic organisms as parts of other metabolic pathways so the presence of any of these enzymes is not considered as a proof of autotrophic capability of an organism but when most of these genes are tracked in a genome, the possibility of autotrophic lifestyle would be considered. In our model, all but 2 of reactions involved in rTCA could be part of other metabolic pathways. These two reactions are catalyzed by aconitate hydratase and citrate-CoA ligase. We set these reactions’ bounds to zero in our mode unless when the possibility of autotrophic growth was of our concern. All other reactions were considered as part of the metabolic network.

## Discussion

**Fig 4:**
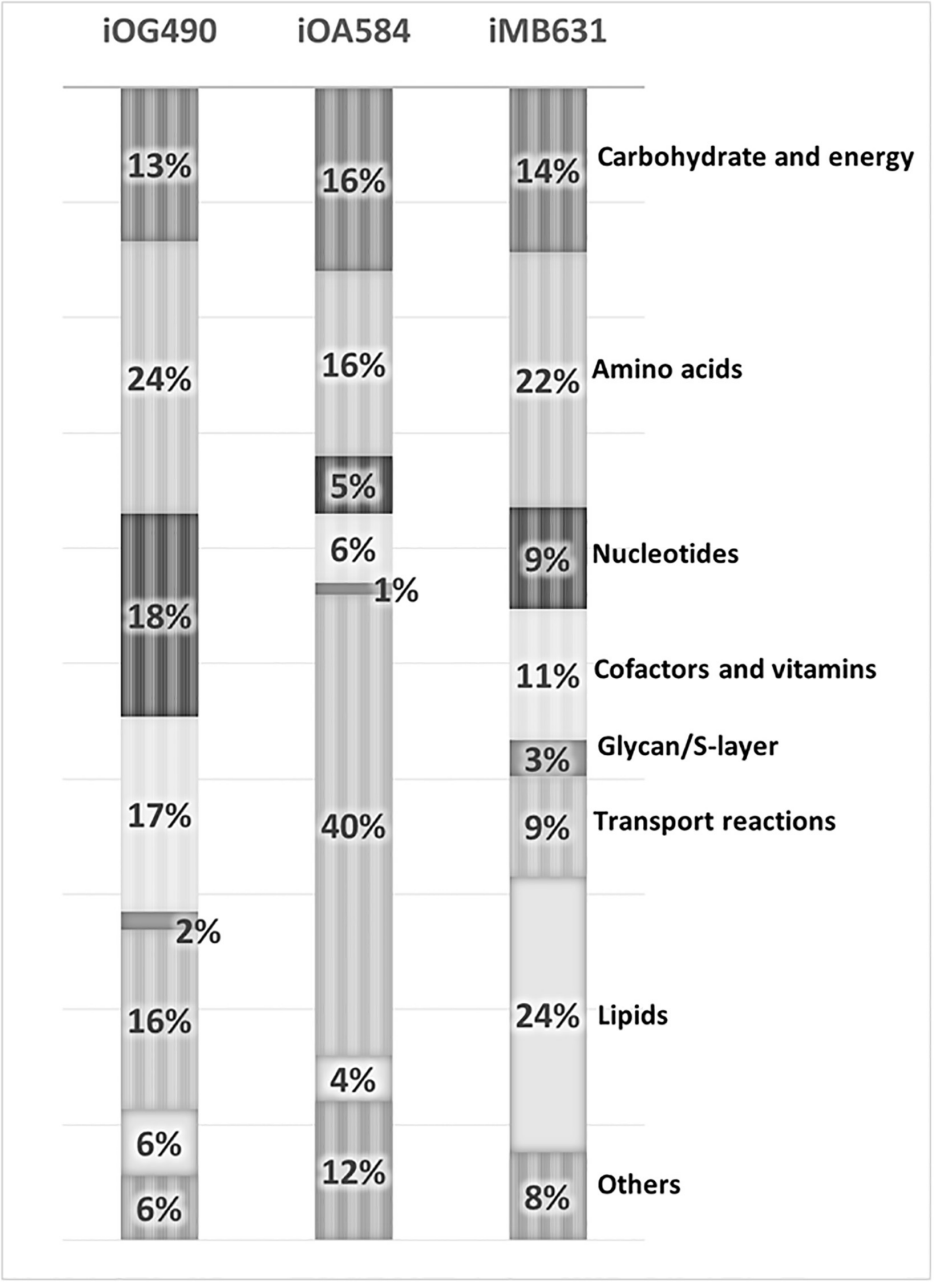
Comparison of reaction distribution in subsystems in three halophilic modes; *i*MB631, *i*OA584, *i*OG490. Amino acid metabolism has the largest number of reactions in both extremophilic models but in moderate halophilic *i*OA584, transport reaction category is the major one, mostly used to import osmo-protectants and related compounds whereas peptidoglycan or S-layer biosynthesis has the smallest set in all three models.

The reactions involved in *i*MB631 were assigned into eight main subsystem based on their functional roles. For comparison purpose, the same categorization was adopted for metabolic models of extremely halophilic archaeon *Halobacterium salinarum* and moderately halophilic bacterium *Chromohalobacter salexigens*, *i*OG490 and *i*OA584 respectively [25, 50]. The results of this comparison are illustrated in Fig 4. The lipid metabolism is the largest subsystem including 24% of *i*MB631 reaction while peptidoglycan metabolism with only 3% of reactions, has the smallest number of related reactions. Amino acid metabolism is more active in *Salinibacter* and *Halobacterium* than *Chromohalobacter*. These two organism were shown to prefer amino acids rather than carbohydrate utilization in laboratory tests and this characteristic is reflected in the models by an increase in the portion of this subsystem in their metabolic models (That is, 22%, 24% and 16% of reactions in *i*MB631, *i*OG490 and *i*OA584 models, respectively). The larger ration of transport reactions in *i*OA584 (40%) in comparison to *i*MB631 and *i*OG490 (9% and 16% respectively) is presumably related to the differences among these organisms’ osmo-adaptation mechanism. *Salinibacter*, like *Halobacterium* and other extremely halophilic archaea, is shown to use the salt-in mechanism which does not include uptake and accumulation of osmolytes. *Chromohalobacter*, in contrast, is shown to uptake and use different organic osmolytes such as betaine, ectoine and *etc*. This moderately halophilic organism can also utilize many carbon sources which is not the case for *Halobacterium* and *Salinibacter*. Transport reactions to import these carbon sources for further utilization is also a reason for the increased ratio of transport reactions in *i*OA584. Lipid metabolism comprises 24% of reactions in our model which is the highest in three compared models for this subsystem.

Our model showed a better function dealing with false positives than false negative results. A higher amount of incorrect predictions in false positive results, *i*. *e*. those with a negative results in experimental tests which incorrectly appeared to be positive in the model, is observed in comparison to false positive results. This can be discussed in the light of previous findings that some enzymes in *Salinibacter ruber* has salt-dependent activity [13, 15, 41]. In other words, the enzymes may exist in the organism but are not active in *S*. *ruber*’s optimal growth salinity (23% total salt) which is routinely used for growth and experimentation of *Salinibacter*.

**Fig 5:**
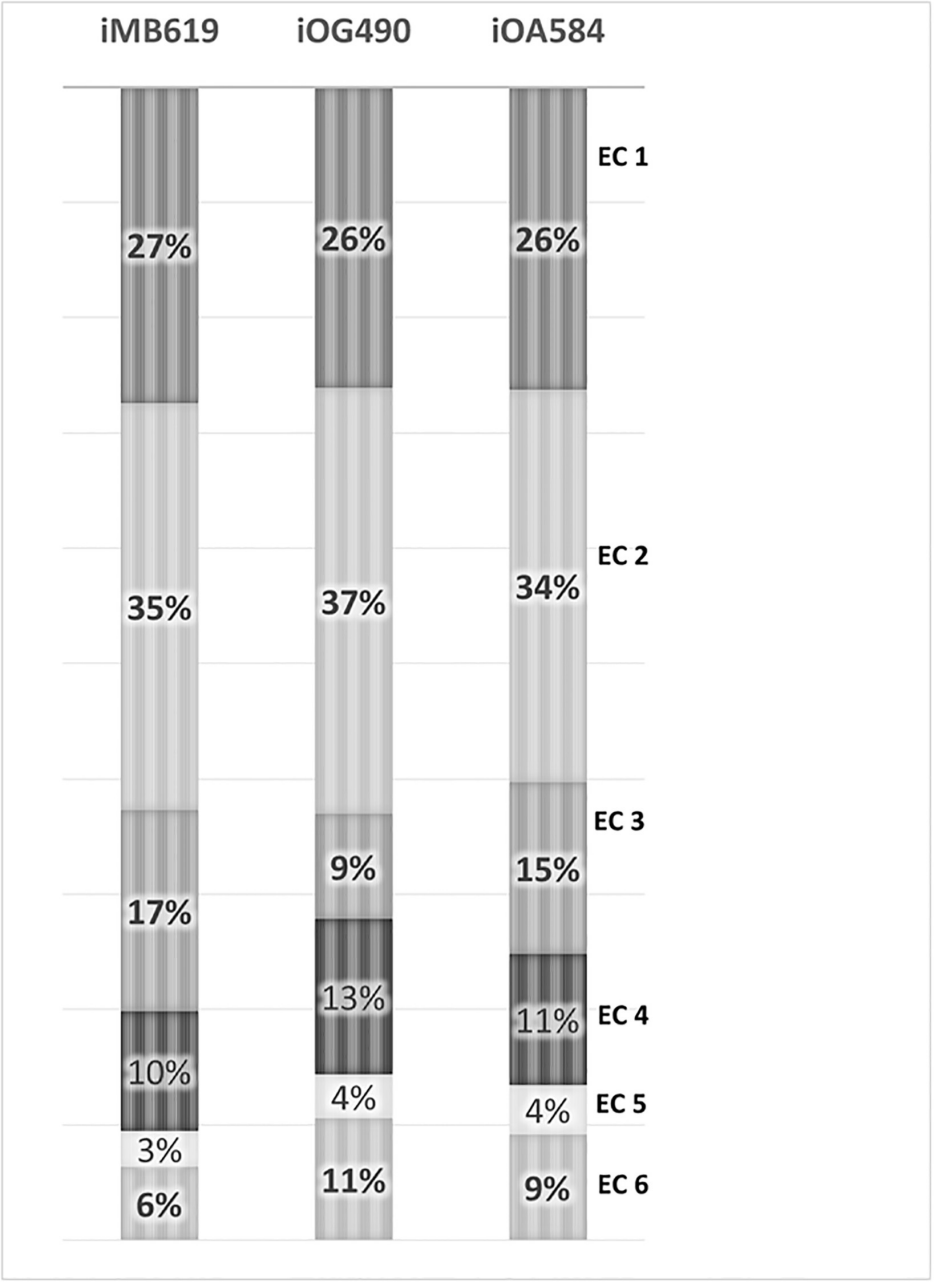
Enzyme classes in *i*MB631, *i*OA584 and *i*OG490. Transferases (EC 2) are the main category of enzymes in all three models whereas isomerases (EC 5) comprise the smallest set. Category of hydrolase (EC 3) enzymes depleted considerably in the archaeal model in comparison to bacterial types.

Distribution of genetically coded enzymes in different enzyme classes is shown and compared in Fig 5. In all three compared model, transferases (EC 2) are the primary enzymes followed by oxidoreductases (EC 1). Hydrolases (EC 3), lyases (EC 4), ligases (EC 6) and isomerases (EC 5) are subsequent in both bacterial models, *i*MB631 and *i*OA584. In the archaeal halophile’s model, *i*OG490, hydrolase (EC 3) enzymes depleted considerably. It should be noted that many reactions in the models are assigned with more than one EC number in KEGG. For example rxn00154 (KEGG reaction ID R00209) in our model is carried out with E1 component (EC 1) and E2 component (EC 2). In such cases, 2 reactions were considered in total counting, each one with an individual EC number.

Considering the FBA analysis of glucose consumption, it seems that pentose phosphate pathway is the active pathway when glucose uptake is activated. Though Embden–Meyerhof glycolytic pathway is present in *i*MB631, overall flux distribution of reactions does not allow it to consume glucose and produce pyruvate in any of the tested fluxes therefore the model predicts that glucose is metabolized mainly through pentose phosphate pathway.

An interesting outcome of our model was the prediction of autotrophic life possibility. By including rTCA related reactions in the model, an acceptable biomass production was achieved when autotrophic growth conditions where simulated. As far as we know, no biological data has ever been reported so we put lower and upper limits of zero to suppress two pathway specific reactions in *i*MB631. It is not clear whether *Salinibacter* conducts a mixotrophic life style or not but *i*MB631 suggests it as a possibility. Limited number of gene sequences have been reported for citrate-CoA ligase the enzyme related to gap-filled reaction of this pathway and little is known about its structure and gene variation. It is possible that the gene is present but not identified in the genome of *S*. *ruber*. The presence of a gene (SrPYP; SRU_2224) for photoactive yellow protein (PYP) in *Salinibacter*’s genome can be considered as a support to this hypothesis. SrPYP is a blue light photoreceptor with an absorption maximum at 431 nm. PYPs are a family of cytoplasmic photoreceptors mostly found in photosynthetic bacteria [7, 18]. More comprehensive physiological studies are needed to investigate the accuracy of this hypothesis.

Salinixanthin and halocapnine derivatives are among the most attractive metabolites in *Salinibacter* strains. However, the pathway and mechanism through which these compounds are synthetized are not well defined in terms of cofactor usage, stoichiometry and gene-protein-reaction relations. Therefore, modeling the production of these metabolites via genome-scale network will not be reliable. Further studies are required for obtaining a well-defined description of the reactions involved in these pathways.

## Supporting information

S1 File The metabolite and reaction lists of *Salinibacter ruber* DSM13855 metabolic model, *i*MB631. (XLSX)

S2 File The SBML format of iMB631 metabolic model. (XML)

S3 File The complete list of genes and related data of *Salinibacter ruber* DSM13855 metabolic model, *i*MB631. (XLSX)
